# Extraintestinal Manifestations of Crohn’s Disease in the Form of Pulmonary Nodules: A Case Report

**DOI:** 10.7759/cureus.7161

**Published:** 2020-03-02

**Authors:** Chandi Garg, Isha Shrimanker, Siddharth Goel, John Mclaughlin, Vinod Nookala

**Affiliations:** 1 Internal Medicine, University of Pittsburgh Medical Center (UPMC) Pinnacle, Harrisburg, USA; 2 Gastroenterology, University of Pittsburgh Medical Center (UPMC) Pinnacle, Harrisburg, USA

**Keywords:** crohn’s disease, inflammatory bowel disease, granuloma, ground-glass opacity

## Abstract

Crohn's disease is a systemic illness with a plethora of extraintestinal manifestations affecting various organs, of which the lungs are relatively rare. Pulmonary involvement may include airway diseases, lung parenchymal diseases, pleural diseases, or drug-related diseases. Tracheobronchial involvement is the most common respiratory presentation, whereas Crohn's disease-related interstitial lung disease is seen less frequently.

A 41-year-old woman with a past medical history of Crohn's disease (status-post subtotal colectomy) presented to the hospital for an enlarging ground-glass opacity in her right middle lobe detected on routine computed tomography of the abdomen six months earlier. The opacity had increased in size from 21 x 18 mm to 28 x 18 mm and another ground-glass opacity in the right lower lobe increased in size from 5 mm to 12.4 mm. A robotic right middle lobectomy with lymph node dissection was done and bronchoscopy showed benign nodular lymphoid hyperplasia and a single perivascular epithelioid granuloma. A year later, her relapsing episodes of cough and shortness of breath were managed with prednisone, 20 mg, for a probable pulmonary manifestation of Crohn's disease. A non-contrast computed tomography of the chest showed interval resolution of the right lower lobe ground-glass opacity. A year after that, she presented to the hospital with increasing cough, shortness of breath, and a new right lower lobe ground-glass opacity (14 x 14 mm) on non-contrast computed tomography of the chest and has been managed with steroids with consideration of immunosuppression.

In conclusion, pulmonary manifestations of Crohn's disease present in a myriad of varieties and often present confounding diagnostic problems necessitating an extensive work-up. Thus, Crohn's disease should be kept in the differential list in case of unusual clinical symptoms and radiological signs of idiopathic pulmonary presentations. These infrequent, and sometimes life-threatening, extraintestinal manifestations need to be considered when dealing with Crohn's disease to avoid further impairment of health status and alleviate patient symptoms by prompt recognition and treatment.

## Introduction

Crohn's disease (CD) is a granulomatous systemic disorder, a type of inflammatory bowel disease (IBD), of unknown etiology. However, 25% of patients with CD may also have extraintestinal manifestations (EIMs) [1]. Uveitis, conjunctivitis, arthritis, erythema nodosum, pyoderma gangrenosum, and primary sclerosing cholangitis are the most common EIMs. The lungs are not classically affected, although there is growing evidence for pulmonary involvement in CD [2]. CD can involve the tracheobronchial tree, the lung parenchyma, and the pleura (Hussein G, Sarao MS, Nookala V, McLaughlin JP: P2265 - Nuisance Neighbors: Gut-Lung Cross Talk in a Case of Crohn’s Disease. Presented at the American College of Gastroenterology 2018 Annual Scientific Meeting, Philadelphia, Pennsylvania, Oct. 9, 2018. http://www.eventscribe.com/2018/ACG/ajaxcalls/PosterInfo.asp?efp=RFNSWFFHSFY2NDI0&PosterID=161364&rnd=9.315258E-02). Latent pulmonary impairment and subclinical alveolitis, as evidenced by lymphocytosis in bronchopulmonary lavage (BAL), are well-recognized [3].

The possible mechanism of lung involvement may include the same embryological origin of the lung and gastrointestinal tract by the ancestral intestine, similar immune systems in the pulmonary and intestinal mucosa, the presence of circulating immune complexes and autoantibodies, and the adverse pulmonary effects of some drugs [4-6].

## Case presentation

A 41-year-old woman with a past medical history of Crohn's disease (CD) (status-post subtotal colectomy) presented to the hospital with three weeks’ duration of left-sided abdominal pain. She had no history of smoking or asbestos exposure. Non-contrast computed tomography (NCCT) of the abdomen and pelvis with coronal reconstruction showed a simple hepatic cyst (left lobe) and no recurrence of CD. She was in remission and not on any immunosuppressants.

**Figure 1:**
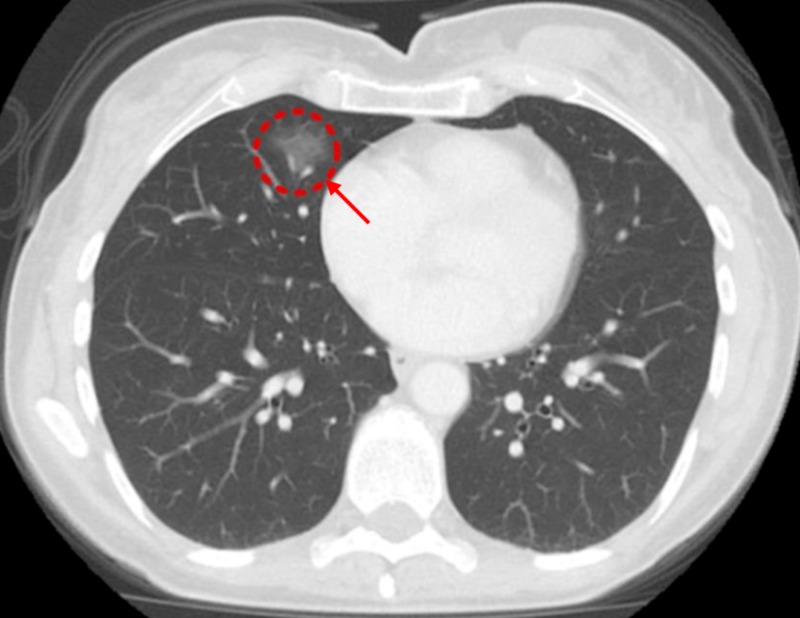
Initial computed tomography (CT) of the abdomen and pelvis with contrast showing partial visualization of ground-glass opacity (GGO) in the right middle lobe (RML)

She was seen six years later for a CD workup and underwent a routine NCCT of the abdomen. The NCCT of the abdomen showed partially visualized, nonspecific, alveolar ground-glass opacity (GGO) in the right middle lobe (RML) (Figure 1).

**Figure 2:**
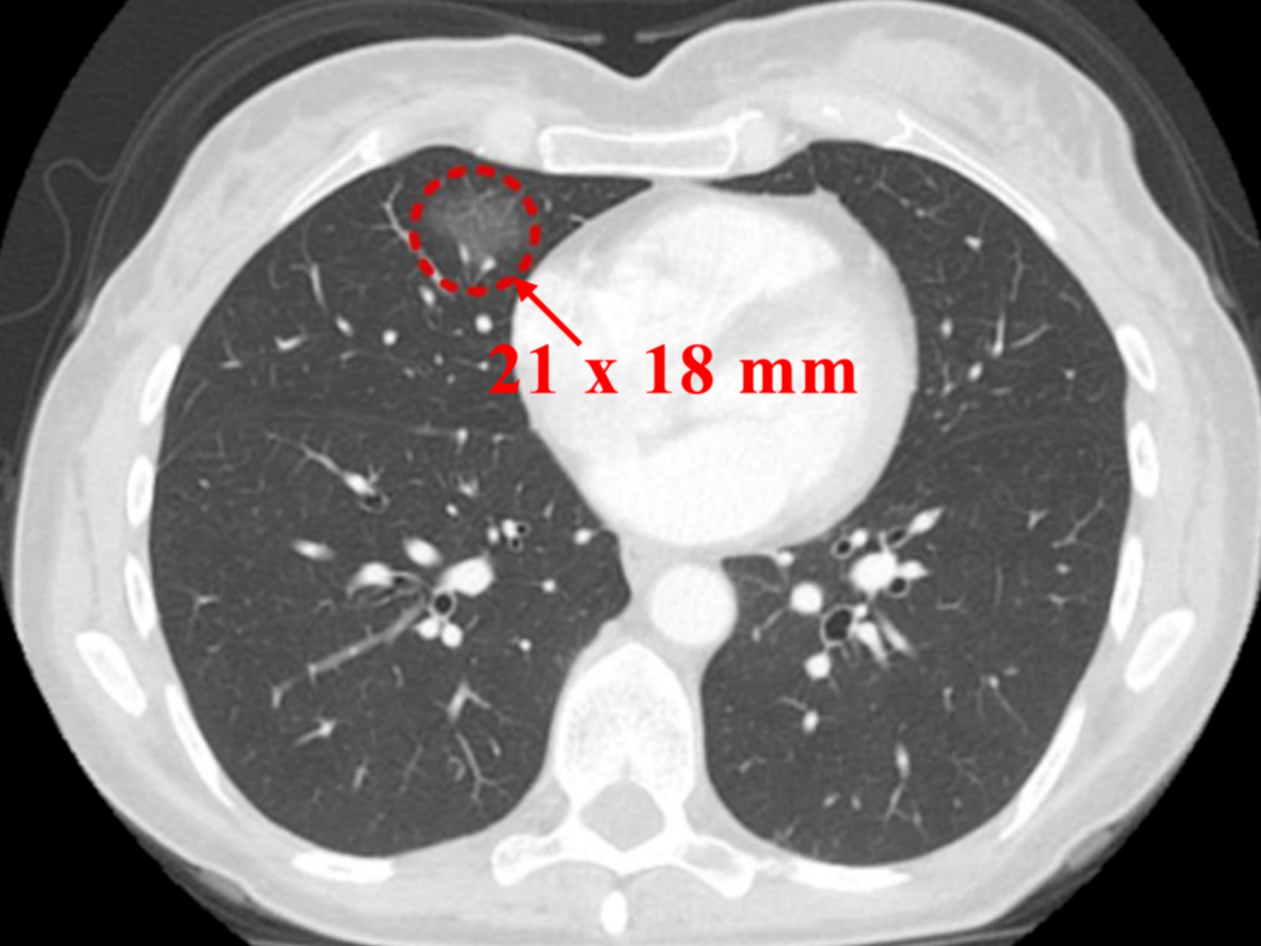
Contrast-enhanced computed tomography (CECT) of the chest (a month following the previous CT)

A month later, a follow-up contrast-enhanced CT (CECT) chest showed 21 x 18 mm enlarging GGO in the RML (Figure 2). With concerns for malignancy, she was admitted to the hospital. She was asymptomatic with a pulse of 62 beats per minute, a blood pressure of 90/70 mmHg, and physical examination was unremarkable. Pulmonary function tests (PFTs) showed forced expiratory volume in one second (FEV1) > 122% and diffusing capacity of the lungs for carbon monoxide (DLCO) > 106%, which were essentially normal.

**Figure 3:**
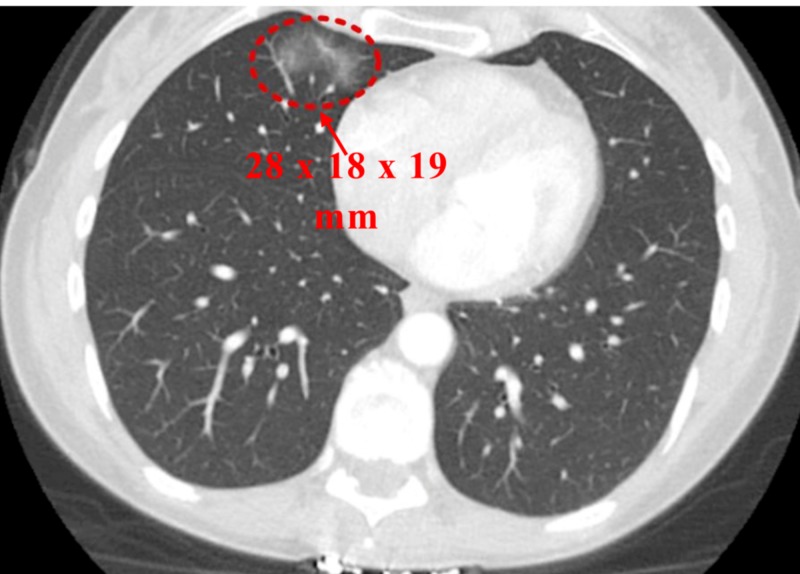
Contrast-enhanced computed tomography (CECT) of the chest (six months from previous CT) Ground glass opacity in right middle lobe increased to 28 x 18 x 19 mm

**Figure 4:**
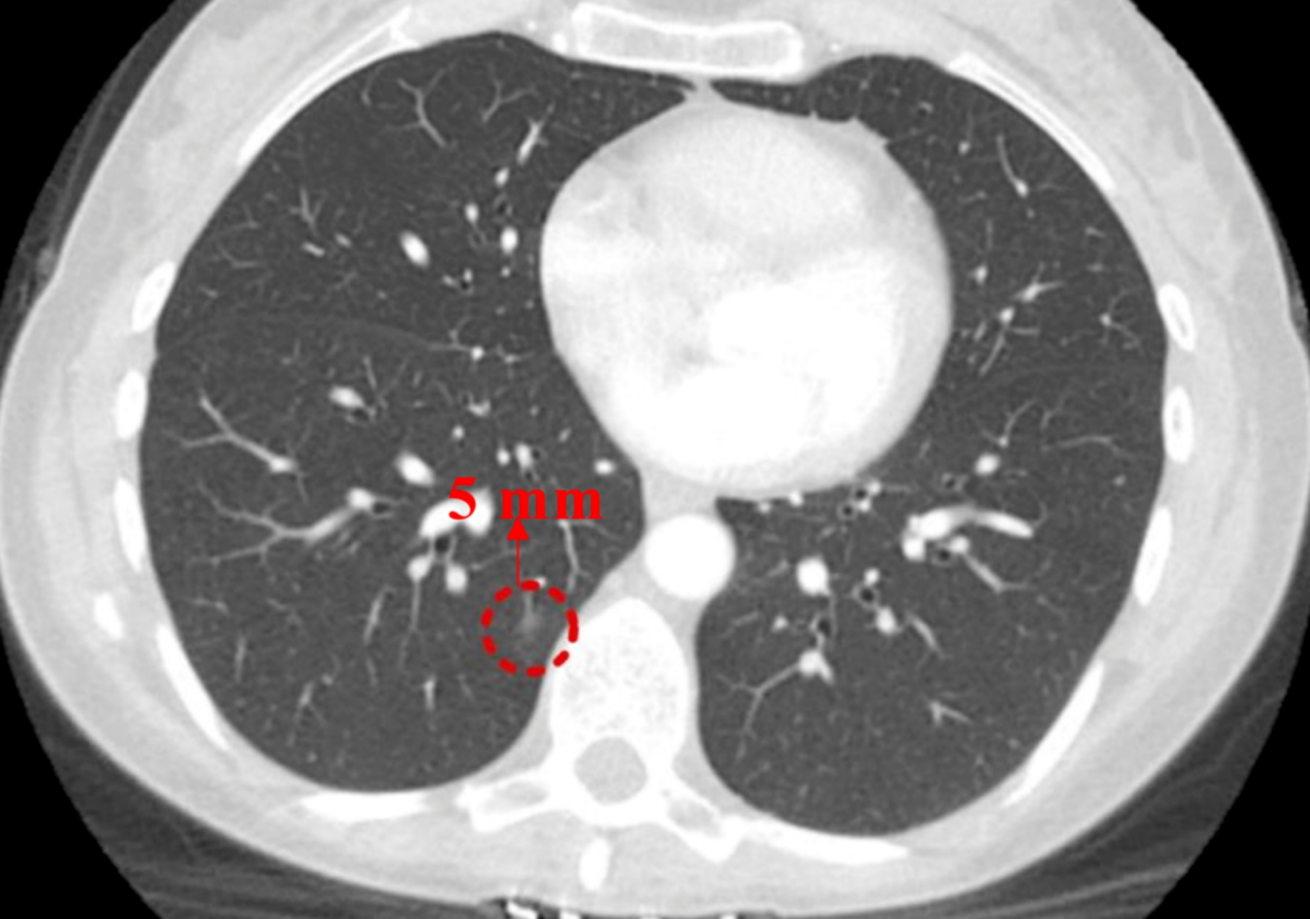
Contrast-enhanced computed tomography (CECT) of the chest (six months from previous CT) New ground-glass opacity (GGO) in the right lower lobe (RLL) measuring 5 mm (appearance concerns for adenocarcinoma with lepidic growth)

A CECT scan six months later showed an increase in the size of the previous GGO (Figure 3) and the appearance of a new GGO measuring 5 mm (Figure 4).

**Figure 5:**
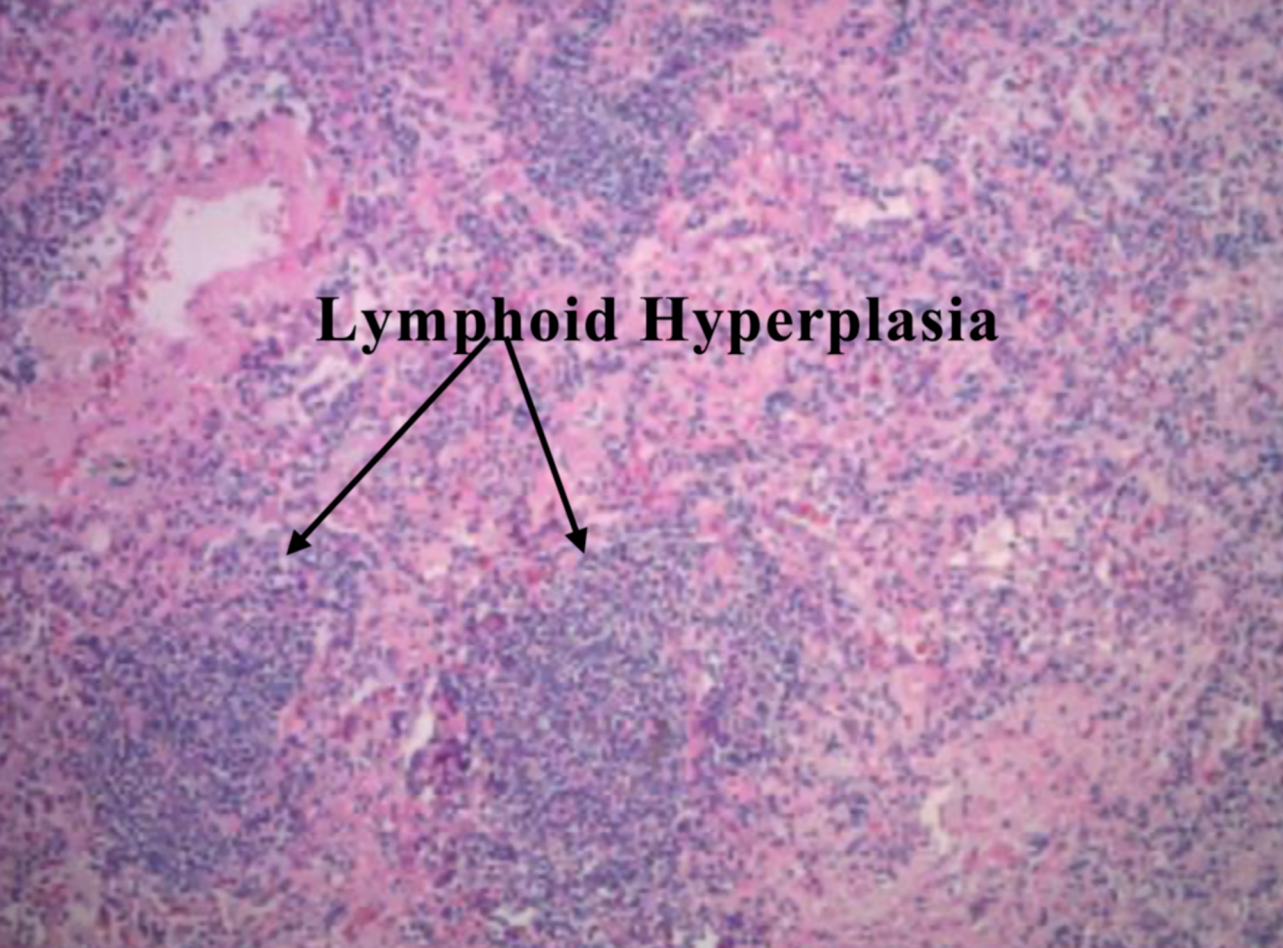
Biopsy of tissue from excised right middle lobe (RML) (one month from previous computed tomography (CT)) (hematoxylin & eosin stain)

She was advised and agreed to a robotic RML lobectomy with lymph node dissection and bronchoscopy for a definitive diagnosis. The biopsy of the tissue sample showed benign nodular lymphoid hyperplasia and a single perivascular epithelioid granuloma (Figure 5).

**Figure 6:**
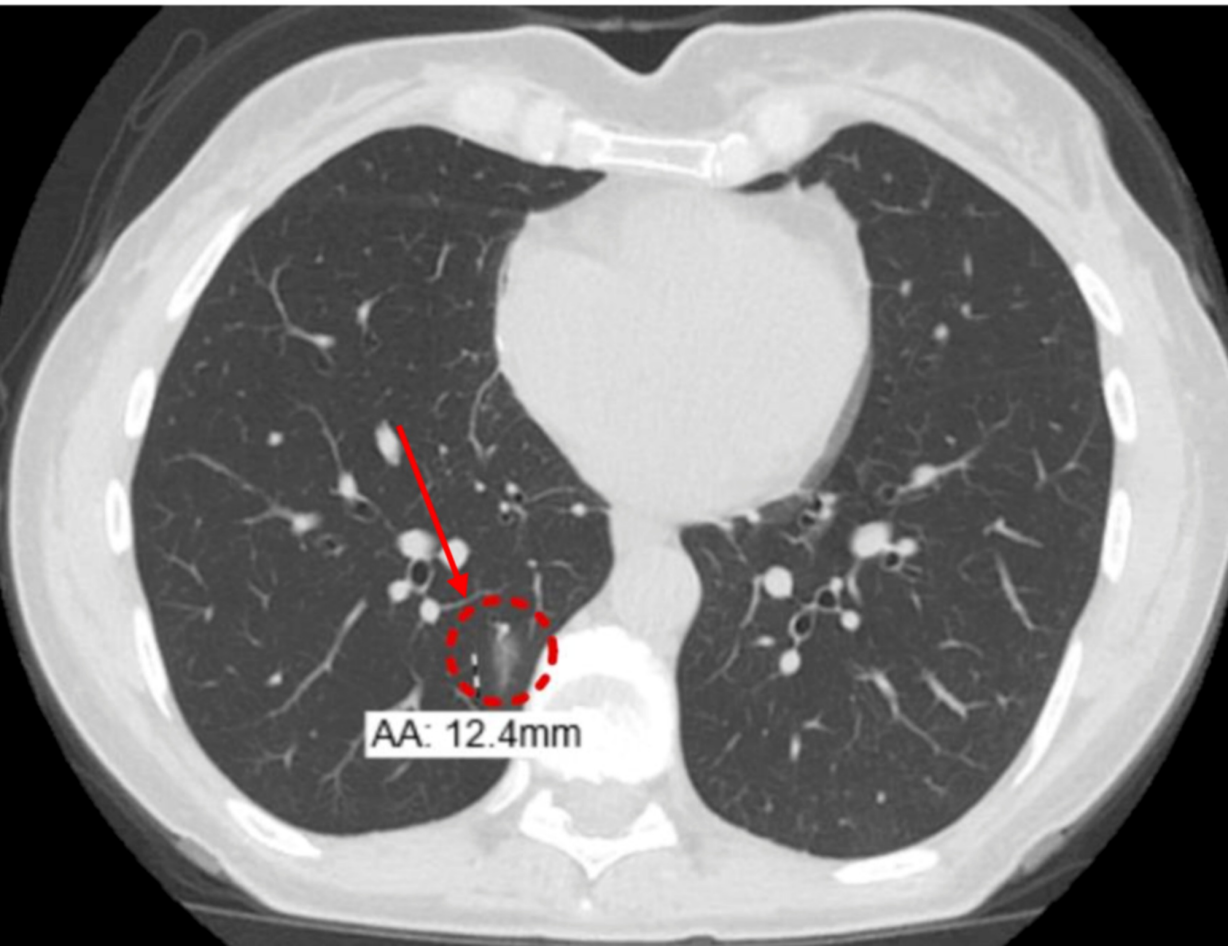
Non-contrast computerized tomography (NCCT) of the chest (eight months from previous CT) Postsurgical changes from right middle lobe resection; enlarging ill-defined 12.4 mm ground-glass opacity in the posteromedial right lower lobe

A month after the surgery, she suffered an episode of Clostridium difficile colitis which was managed with metronidazole and vancomycin (Figure 6). She also complained of a persistent cough.

**Figure 7:**
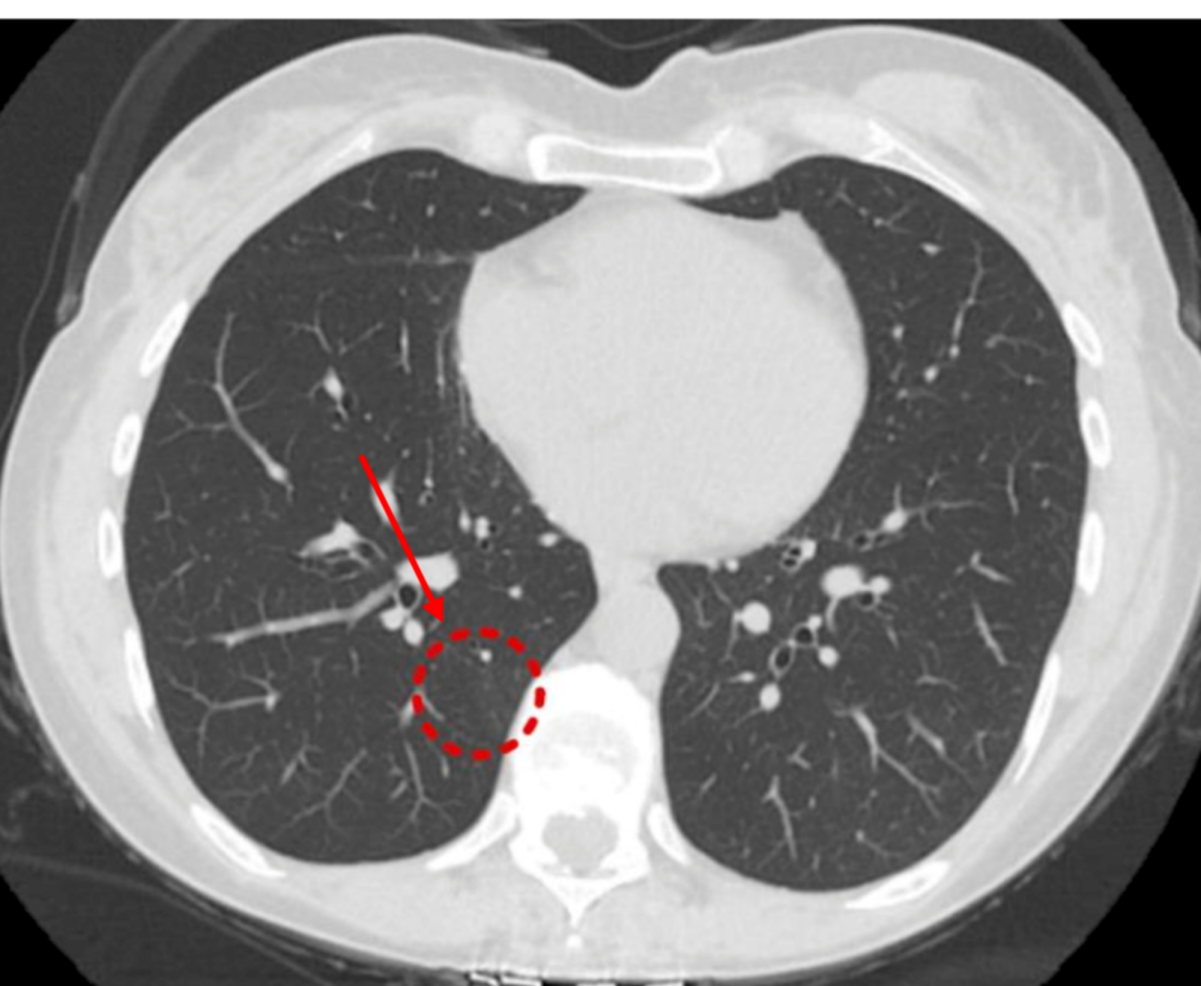
Non-contrast computerized tomography (NCCT) of the chest (three months from previous CT) Interval resolution of ground-glass opacity in the right lower lobe

Nine months post-surgery, she was started on prednisone, 20 mg, for a probable pulmonary manifestation of CD which led to symptomatic relief in her cough and shortness of breath. A follow-up NCCT of the chest, after two months on steroid therapy, showed interval resolution of RLL GGO (Figure 7).

**Figure 8:**
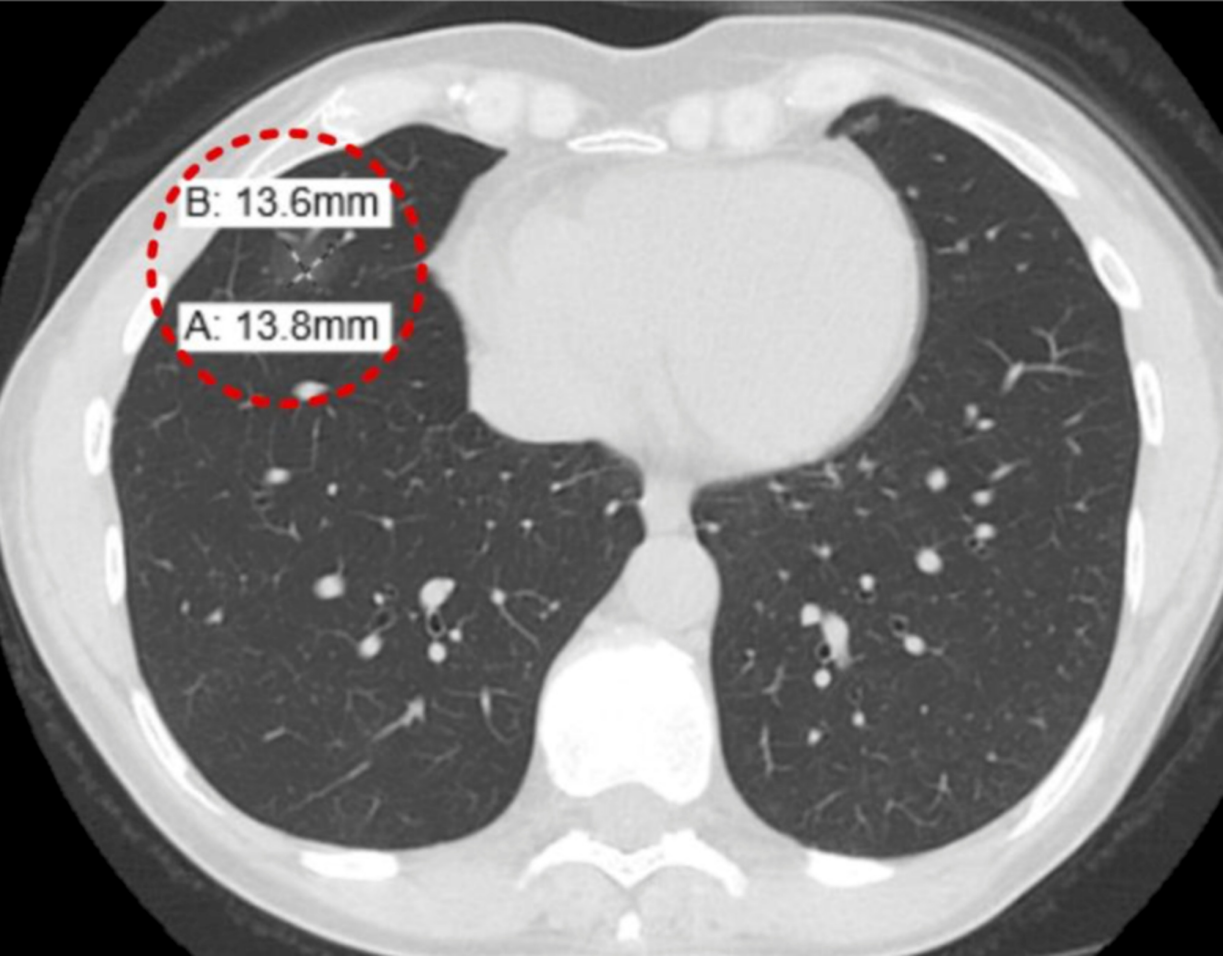
Non-contrast computerized tomography (NCCT) of the chest (10 months from previous CT) New 14 x 14 mm ground-glass opacity in the right lower lobe

After being on steroid therapy for a year, she presented to the hospital for increased cough and shortness of breath. Her vitals were stable, and the examination was unremarkable. An NCCT of the chest showed a new 14 x 14 mm GGO in the RLL (Figure 8).

**Figure 9:**
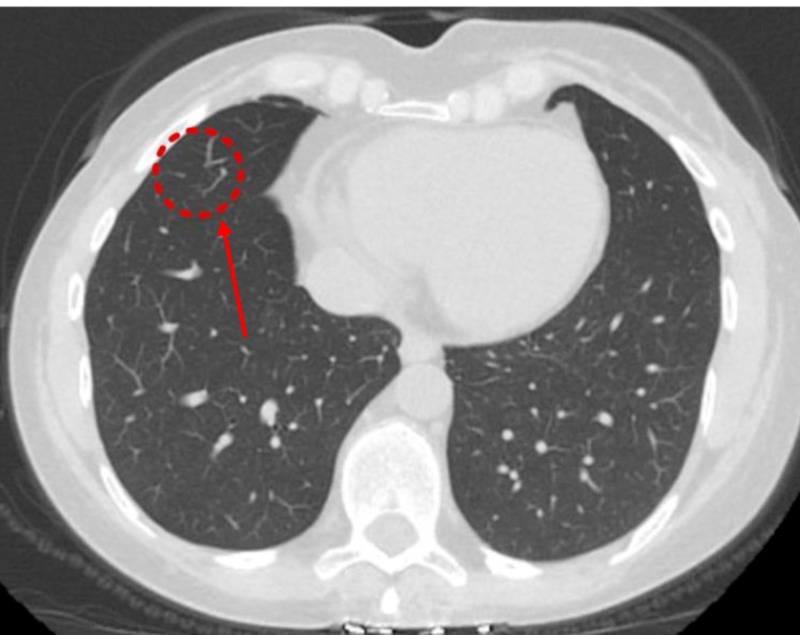
Non-contrast computerized tomography (NCCT) of the chest (two months from previous CT) showed resolution of the previously identified ground-glass opacity in the right lower lobe

A repeat CT scan after two months showed a resolution of previously identified GGO in the RLL (Figure 9). She may require systemic immunosuppression, such as Infliximab, to prevent flare-ups in the future. 

## Discussion

Pulmonary IBD involves different pathological entities. Storch et al. have categorized them by disease mechanism into drug-induced disease, anatomic disease, overlap syndromes, autoimmune disease, physiologic consequences of CD, pulmonary function tests abnormalities, and non-specific lung disease [7]. The diagnosis was based on chest radiograph, CT findings, bronchoalveolar lavage, and characteristic histological features. In IBD, pulmonary abnormalities can occur due to drugs, like sulfasalazine and mesalamine (leading to eosinophilic pneumonia and fibrosing alveolitis) or methotrexate (leading to pneumonitis). Opportunistic lung infections can occur due to immunosuppressive treatment with anti-tumor necrosis factor (TNF) alpha antibodies, azathioprine, or calcineurin antagonists. Therefore, drug-induced lung disease and opportunistic infections must be ruled out before considering EIM of CD. Bronchoalveolar lavage characteristics can be helpful in differentiation with lymphocytic bronchoalveolar lavage found in CD-related interstitial lung disease compared to an eosinophilic bronchoalveolar lavage in drug-induced pulmonary interstitial complications.

Bowel or lung - which comes first?

Interstitial lung disease (ILD) may precede the first symptoms of bowel disease by years, but more commonly, like in our patient, it appears in patients with longlasting CD [8]. In our patient, GGO was seen nine years from the initial diagnosis of the CD. Besides, the signs and symptoms are not related to bowel disease activity and may be present in patients with inactive CD [9]. Asymptomatic lung involvement is possible. Respiratory symptoms, as seen in our patient, include a dry cough, breathlessness on exertion or at rest, and chest tightness. Expectoration of blood-stained sputum is rare. 

Imaging patterns

Imaging in CD patients with pulmonary involvement shows that pneumonia-like opacities are very frequent [2]. Multiple pulmonary nodules of different sizes and locations may be present. Metastatic lung disease becomes a differential if the lesions are coin-shaped and bigger. Subpleural nodes and small nodules may be seen, which may mimic primary lung tumors [9]. Small cavitations reflecting central necrosis may be present. The CT scans in cases with interstitial pneumonitis reveal GGO, alveolar filling, or a reticular pattern [10]. Upper lobe fibrosis may resemble tuberculosis (TB) infection. Extensive fibrosis is rare. Piotrowski et al. reported vanishing patterns of radiological lesions not related to treatment or CD activity [9]. Faller et al. reported spontaneous migration of the lung opacities [11]. In the study by Mahadeva et al., only one patient out of 17 studied had a mixed reticular and ground-glass pattern in the mid zones with a patchy distribution in the central and peripheral portions of the lungs with air trapping [10]. It reflects the low frequency of ILD and the high frequency of tracheobronchial pathology in patients with CD [12]. Recently, Desai et al. reported similar results (Table 1) [2].

**Table 1:** Possible Pathological Patterns of Crohn's Disease-related Interstitial Lung Disease Based on published case reports [16]

Pathological pattern	Frequency
Non-specific lymphocytic infiltrations	High
Organizing pneumonia	High
Non-caseating granulomas	High
Eosinophilic pneumonia	Moderate
Necrobiotic pulmonary nodules	Moderate
Amyloid nodules	Incidental
Necrotizing granuloma	Incidental

Notably, most of the patients with a CT abnormality are respiratory symptom-free [12].

Tests

PFTs may show a restrictive pattern [13-14]. DLCO may be decreased, or normal [11]. Hypoxemia at rest or on exertion may be present [13. Munck et al. and Ateş et al. found that respiratory symptom-free patients have decreased DLCO during exacerbations of the bowel disease [15-16]. Bronchial hyperresponsiveness has been demonstrated in 27% of CD patients [17]. About 20% of the patients with pulmonary involvement have dormant bowel disease suggesting latent asymptomatic lung inflammation in CD patients [12]. 

Diagnosis is usually confirmed by surgical lung biopsy, but transbronchial peripheral lung biopsy may be sufficient [11, 13]. Therefore, it is preferred to have a less invasive method.

Prognosis

Overall, the prognosis is good as it responds well to treatment with systemic steroids, as supported by normalization of PFTs after treatment. Aerosolized, oral, or intravenous steroids are effective in treating the clinical symptoms of pulmonary involvement. Moderate doses of 0.5 - 1.0 mg/kg of prednisone daily with gradual dose reduction are usually sufficient. However, relapses may occur when the steroid dose is tapered down or withdrawn [2, 13]. Rare, severe interstitial lung fibrosing pneumonitis may be steroid-resistant and fatal [18]. Hotermans et al. showed that in more severe cases, the addition of cyclophosphamide to a high dose of steroid treatment improves the outcome [13]. An anti-TNF treatment, such as infliximab, may be a useful alternative or adjunct to steroids [19].

## Conclusions

In conclusion, CD is a systemic disorder and not restricted to the intestines. Pulmonary manifestations of CD can present a confounding diagnostic problem necessitating an extensive work-up. Patients suffering from CD should undergo a pulmonary evaluation which should include physical examination, chest x-ray, and PFTs with measurement of DLCO. Invasive measures, such as bronchoscopy and thoracoscopy, are typically required to reach a final diagnosis and steroids are the most frequently reported treatment. The addition of cyclophosphamide or infliximab to severe or steroid-resistant cases requires further study.
